# Supplementary material for: Multifaceted membrane interactions of human Atg3 promote LC3-phosphatidylethanolamine conjugation during autophagy
Source: Nat Commun. 2023 Sep 7;14:5503. doi: 10.1038/s41467-023-41243-4 (PMC10485044; doi:10.1038/s41467-023-41243-4)
Supplement: Supplementary file 1 — Supplementary Information [file 41467_2023_41243_MOESM1_ESM.pdf]

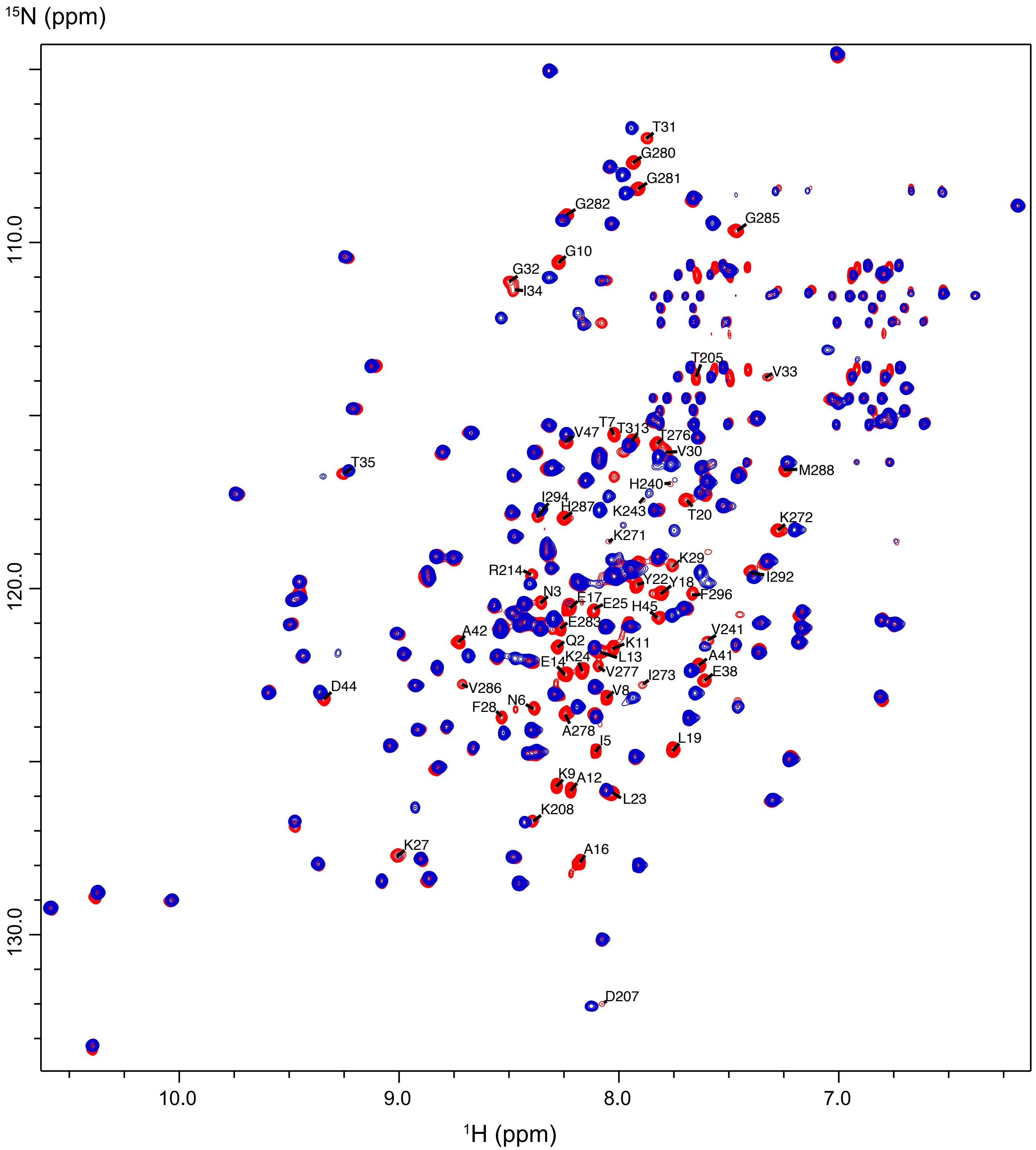

Supplementary Figure 1: Deletion of the first 25 residues of the N-terminal of hAtg3 causes minimal perturbations to its core structure, although some perturbations of C-terminal residues are observed.

Overlay of ^{15}N-^{1}H TROSY spectra of ^{15}N-labeled hAtg3$^{\Delta 90\text{-}190}$ (red) and hAtg3$^{\Delta N25,\ \Delta 90\text{-}190}$ (blue) at pH 6.5. Perturbed residues are indicated.

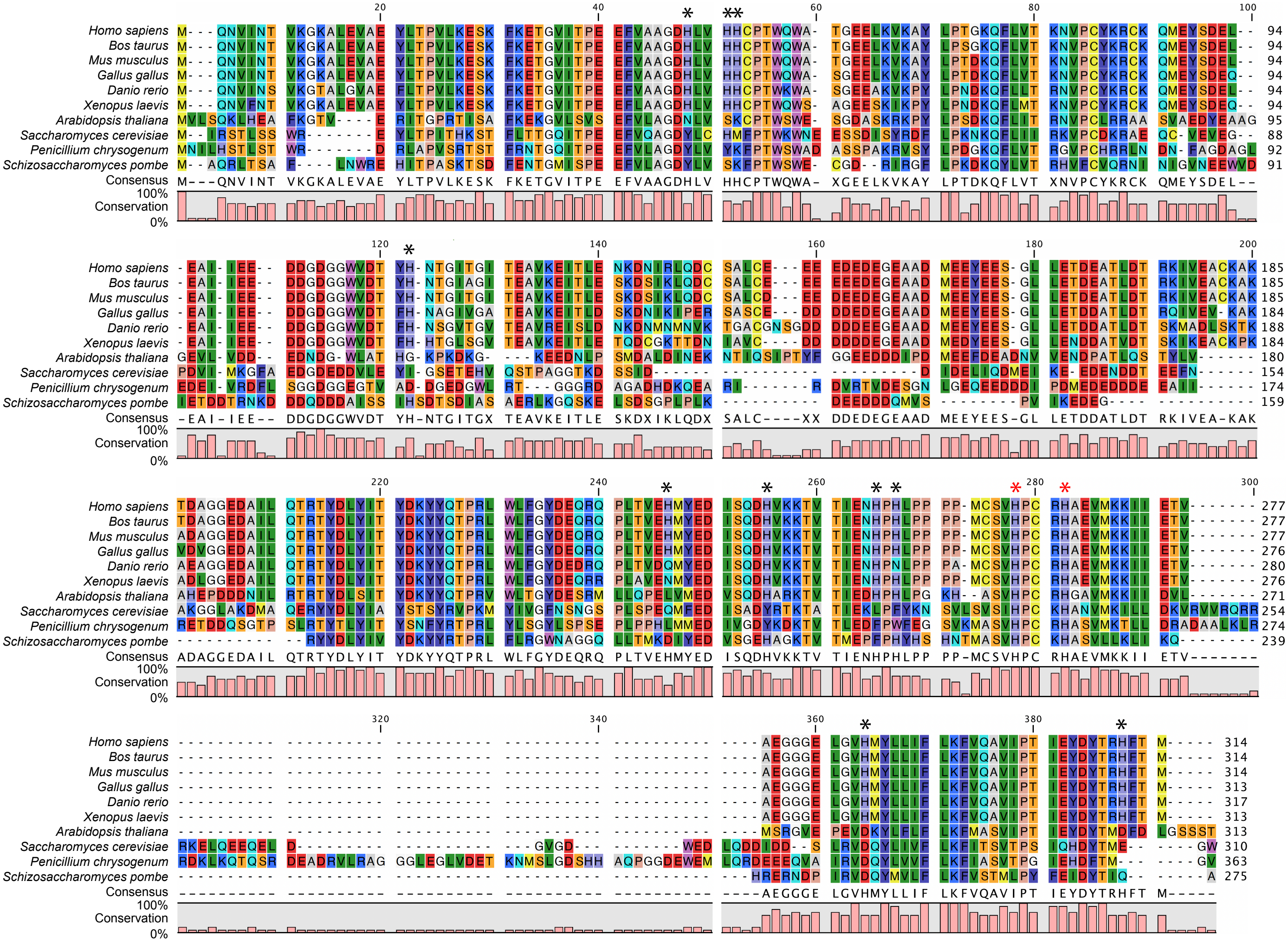

Supplementary Figure 2: Sequence alignment of Atg3 homologs.
His residues of human Atg3 are indicated by asterisks (red asterisks for the conserved His).

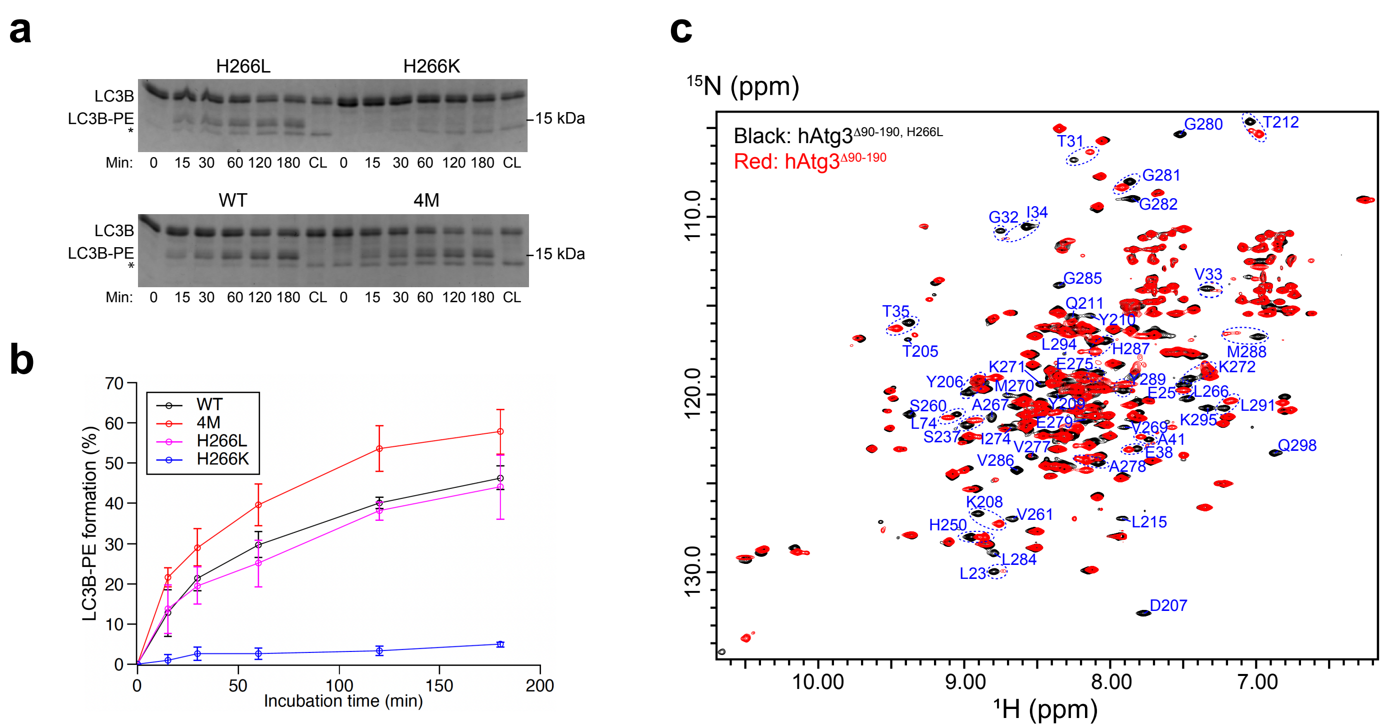

Supplementary Figure 3: H266L mutation stabilizes hAtg3 interaction with bilayer-like bicelles.

(a) SDS-PAGE gel images of time-dependent formation of LC3B–PE for hAtg3 (WT) and H266L, H266K, H240Y/V241A/P263G/H266L (referred to as 4M) mutants. CL represents the control without liposomes at 180 mins. Asterisk indicates a small amount of degradation of LC3B in the presence of ATP. Source data are provided as a Source Data file.

(b) Plots of time-dependent formation of LC3B–PE for hAtg3 and mutants. Data are presented as mean ± SD. Quantification of conjugation reactions was obtained from three separate measurements (n = 3). Source data are provided as a Source Data file.

(c) Overlay of ^{2}H,^{15}N,^{13}C-labeled hAtg3$^{\Delta 90\text{-}190,\ H266L}$ (black) and hAtg3$^{\Delta 90\text{-}190}$ (red) TROSY spectra in bicelles (DMPC:DMPG:DHPC = 4:1:20, molar ratio, q = 0.25). Multiple new resonances in the spectrum of hAtg3$^{\Delta 90\text{-}190,\ H266L}$ were observed and assigned to the hAtg3 catalytic region. Several perturbed residues are circled.

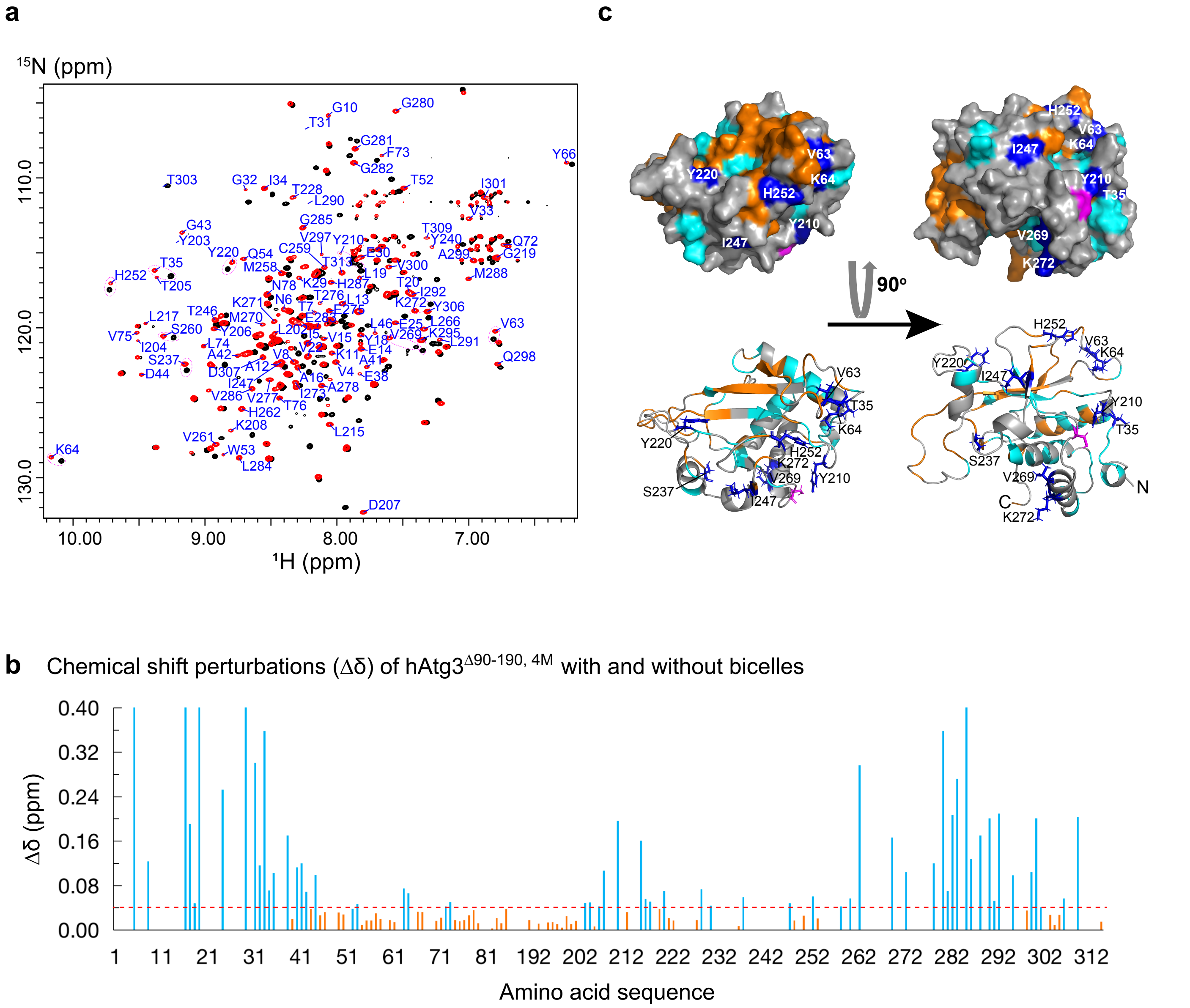

Supplementary Figure 4: hAtg3$^{Δ90-190, 4M}$ binding to bicelles induces structural arrangements in its catalytic region.

(a) Overlay of ^{2}H,^{15}N,^{13}C-labeled hAtg3$^{Δ90-190, 4M}$ TROSY spectra at pH 7.5 in the absence (black) and presence (red) of bicelles (DMPC:DMPG:DHPC = 4:1:20, molar ratio, q = 0.25). Perturbed resonances are labeled with their assignments in bicelle-bound state.

(b) Plots of hAtg3$^{Δ90-190, 4M}$ ^{15}N and ^{1}H chemical shift perturbations (CSPs, Δδ) induced by bicelles against residue numbers. $\Delta\delta = \sqrt{0.5*\{(\delta_N/5)^2 + \delta_H{}^2\}}$, δ_H and δ_N represent the changes in ^{1}H and ^{15}N chemical shifts upon interacting with bicelles, respectively.

(c) Surface and ribbon representations of hAtg3 structure with perturbed residues (Δδ ≥0.04 ppm) colored light blue and unperturbed residues (Δδ < 0.04 ppm) colored orange. The active site Cys264 is highlighted in magenta and uncharacterized residues are colored grey. Some resonances that display large CSPs are circled in **(a)**; their corresponding residues are colored blue in **(c)**.

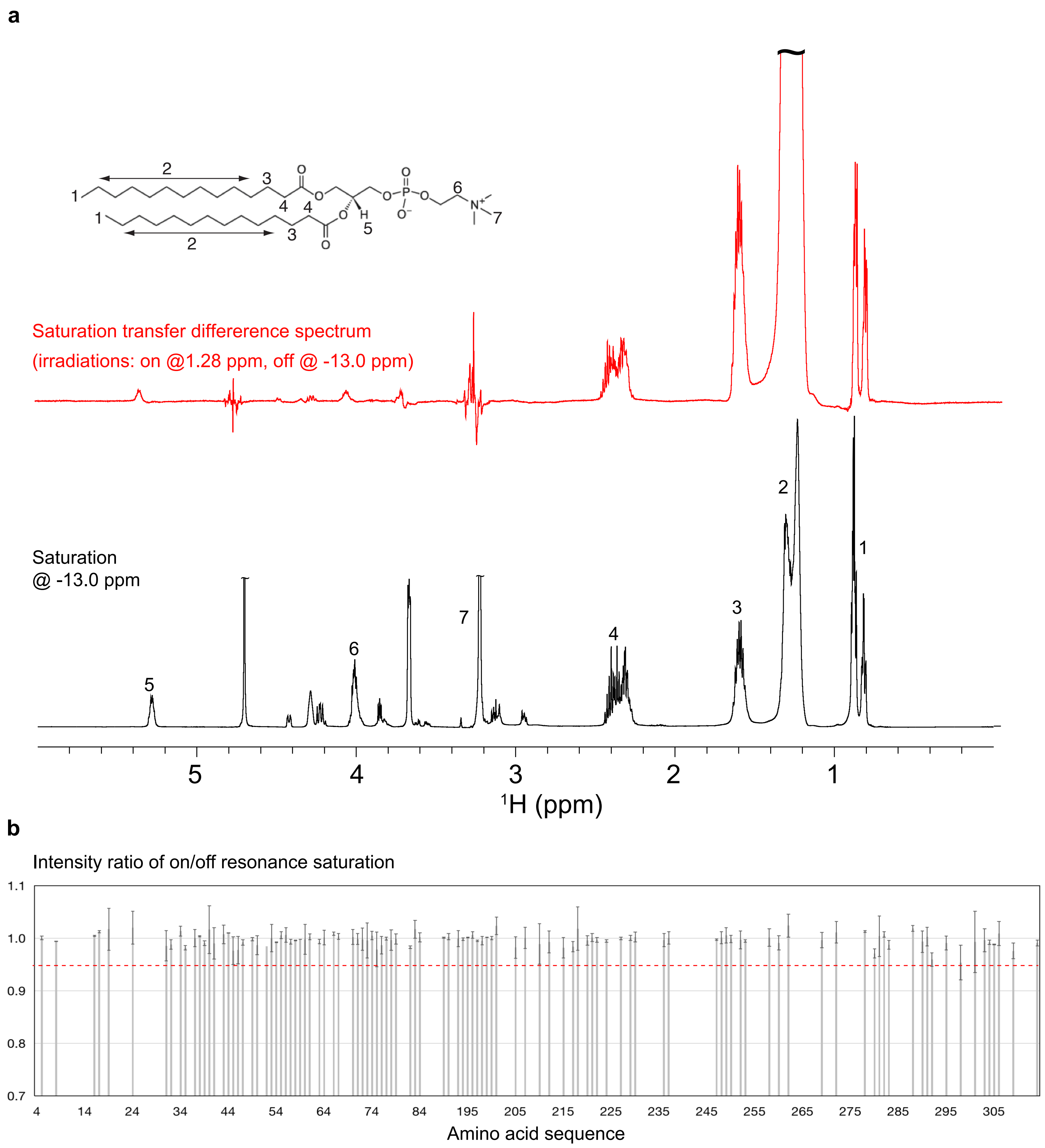

Supplementary Figure 5: NMR cross-saturation experiments.

(a) Effective saturations of lipid resonances by irradiation at 1.28 ppm (peak #2).
Black: 1D ^{1}H spectrum of ^{2}H, ^{15}N, ^{13}C-hAtg3$^{\Delta 90\text{-}190,\ 4M}$ in bicelles with saturation at -13.0 ppm (off).
Red: Saturation transfer difference spectrum of ^{2}H, ^{15}N, ^{13}C-hAtg3$^{\Delta 90\text{-}190,\ 4M}$ in bicelles.

(b) hAtg3$^{\Delta 90\text{-}190,\ 4M}$ in aqueous solution experiences few cross-saturation effects.
Plot of cross saturation effects against residue number for perdeuterated ^{15}N, ^{2}H- hAtg3$^{\Delta 90\text{-}190,\ 4M}$ in an 80% D_2O and 20% H_2O solution with saturations at -13.0 ppm (off) and 1.28 ppm (on).
Source data are provided as a Source Data file.

a

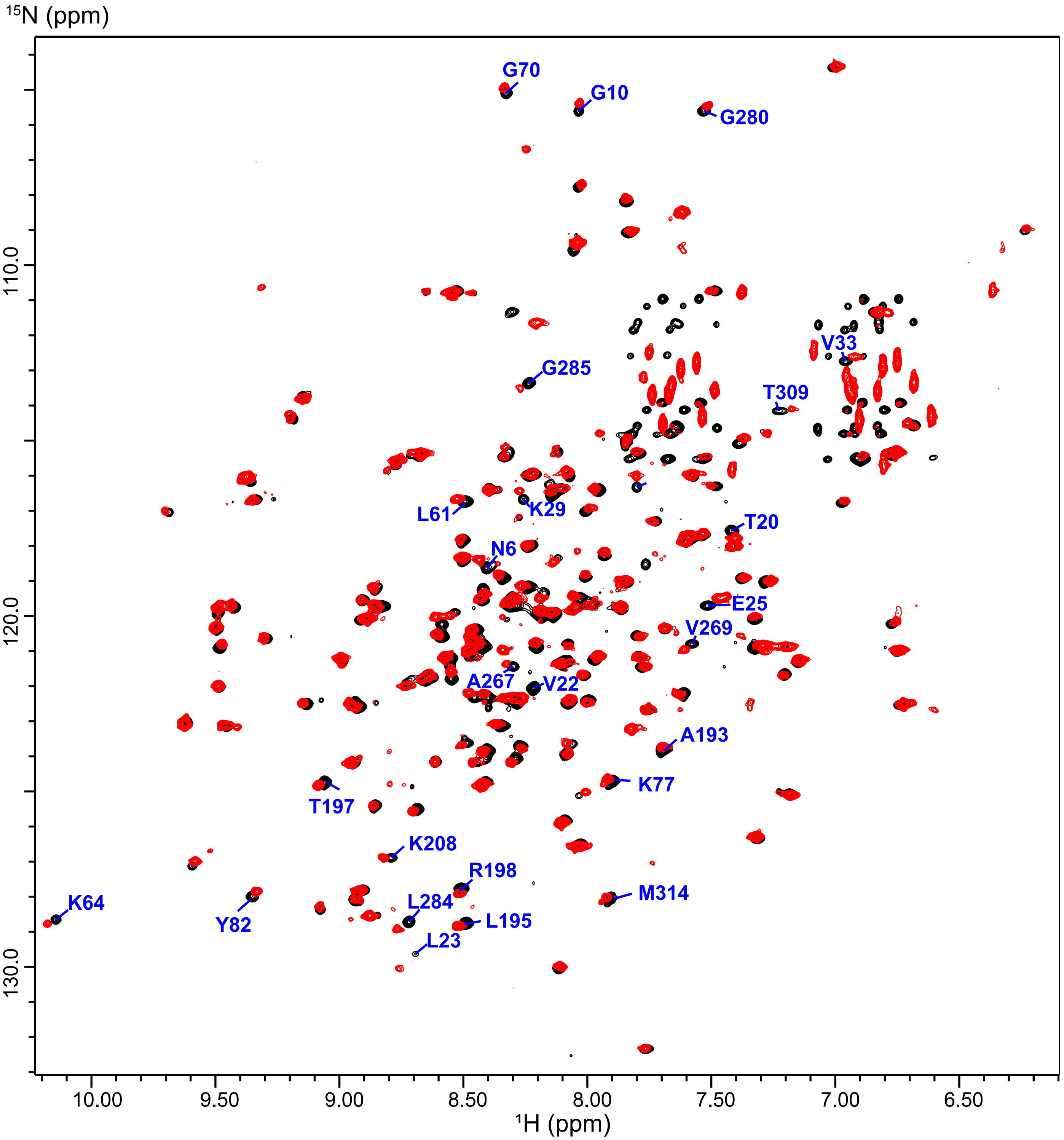

Supplementary Figure 6: LPE lipids cause minor perturbations on the structure of hAtg3$^{\Delta 90\text{-}190,\ 4M}$ in bicelles.

(a) Overlay of ^{2}H, ^{15}N-labeled hAtg3$^{\Delta 90\text{-}190,\ 4M}$ TROSY spectra in DMPC:DMPG:DHPC = 8:2:20 (molar ratio) bicells (black) and DMPC:DMPG:LPE:DHPC = 7:2:1:20 (molar ratio) bicelles (red).

b

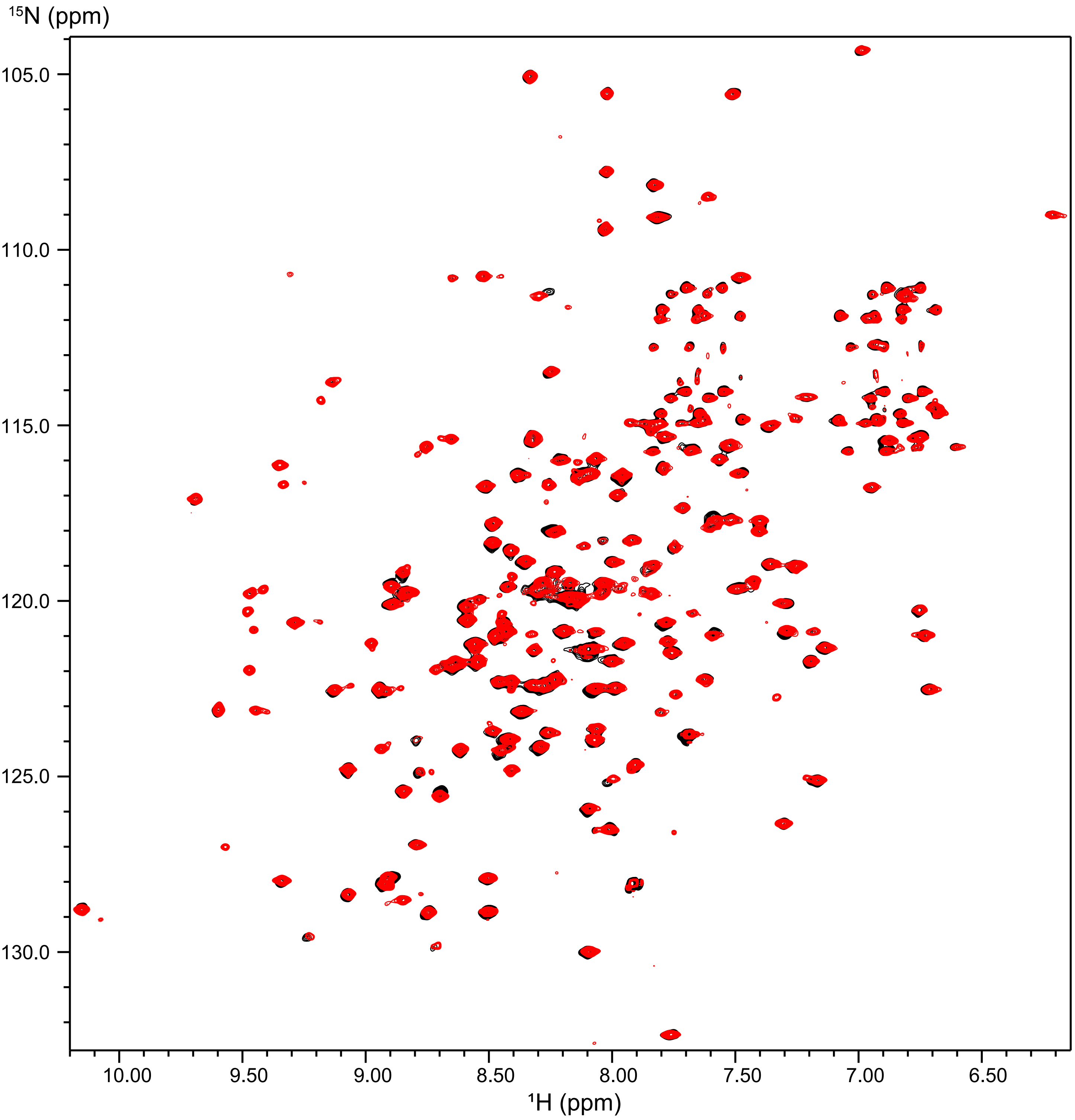

Supplementary Figure 6:

(b) Overlay of ^{2}H, ^{15}N-labeled hAtg3$^{\Delta 90\text{-}190,\ 4M}$ TROSY spectra in DMPC:DMPG:LPC:DHPC = 7:2:1:20 (molar ratio) bicells (black) and DMPC:DMPG:LPE:DHPC = 7:2:1:20 (molar ratio) bicelles (red).

All spectra were acquired on a 600 MHz Bruker spectrometer at 25 °C, pH 7.5. Some perturbed residues are labeled with their assignments.

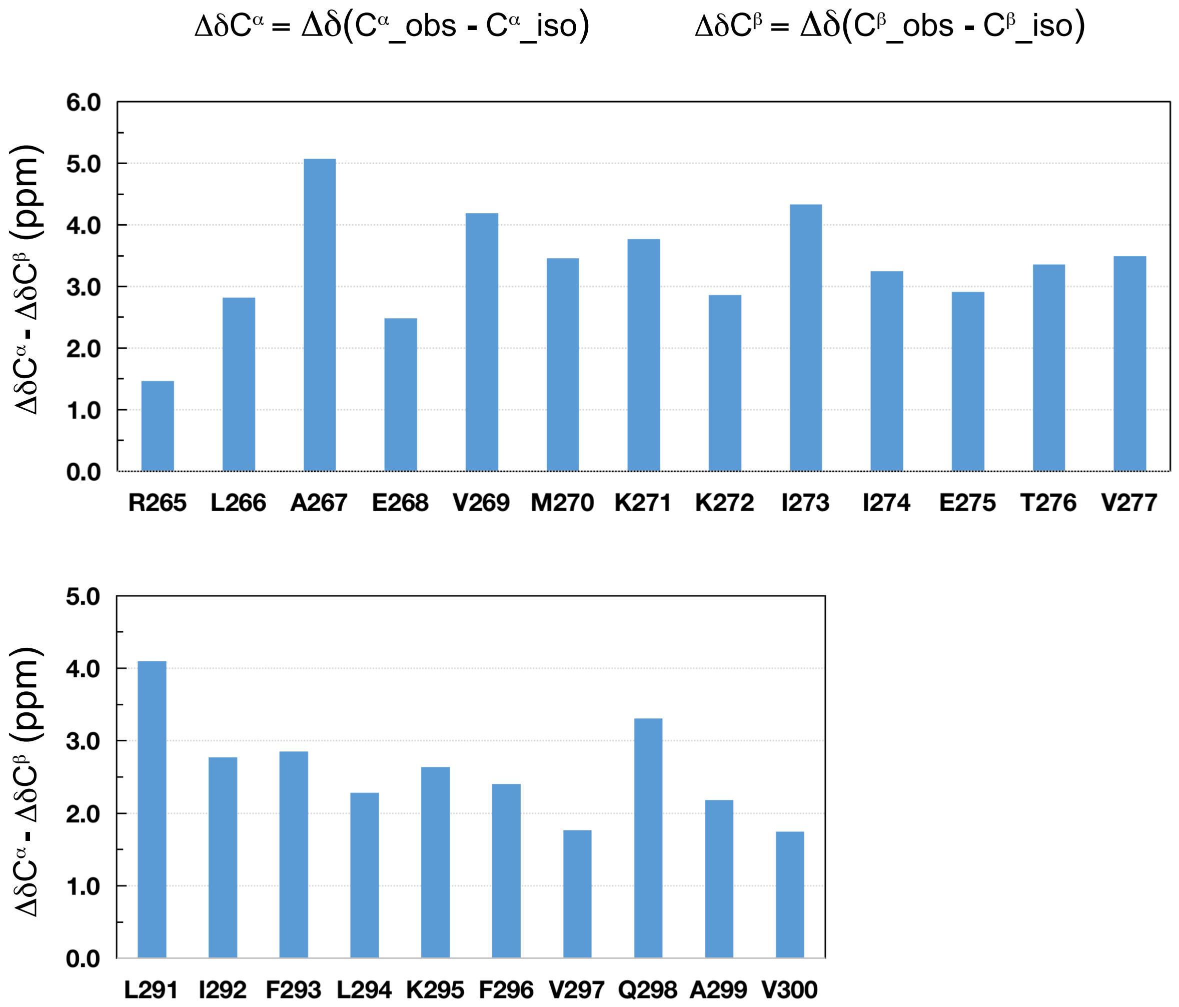

Supplementary Figure 7: $^{13}C^{\alpha}$ and $^{13}C^{\beta}$ secondary chemical shifts indicate helical structures for residues 265 to 277 and residues 291 to 300 in bicelle-bound hAtg3$^{\Delta 90\text{-}190,\ 4M}$. $^{13}C^{\alpha}$_obs and $^{13}C^{\beta}$_obs are observed chemcial shifts while $^{13}C^{\alpha}$_iso and $^{13}C^{\beta}$_iso are values for random coils (Lukin et al., J. Biomol. NMR, (1997), 9: 151-166; Mielke et al., J. Biomol. NMR (2000), 30: 143-153). Deuterium isotope shifts were not corrected. Source data are provided as a Source Data file.

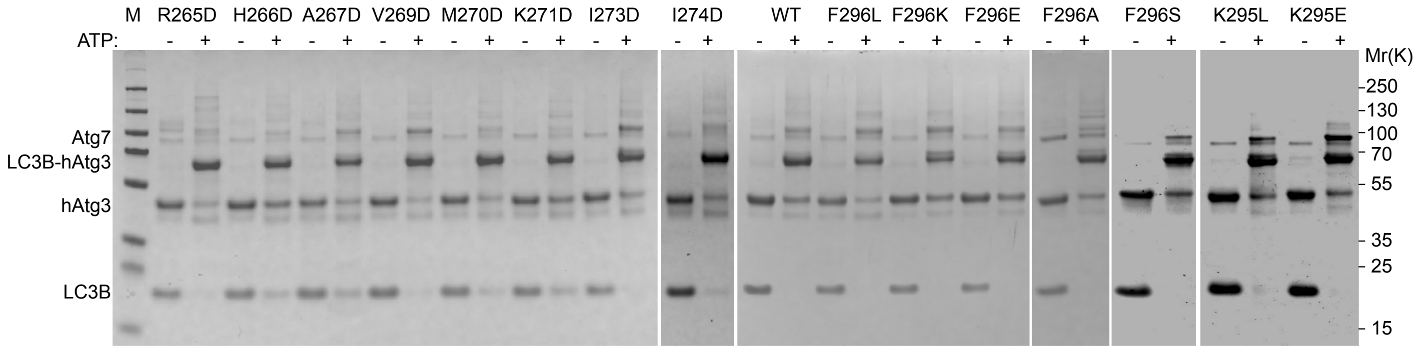

Supplementary Figure 8: hAtg3 mutants form the LC3B-hAtg3 intermediate normally.

SurePAGE (10% Bis-Tris, GenScript) images of hAtg3 wildtype (WT) and its mutants (5 μM) incubated with mouse Atg7 (0.5 μM), LC3B (5 μM) with (+) and without (-) ATP (1 mM) for 30 min at 37 ^{o}C. The formation of intermediate LC3B-hAtg3 is indicated. All experiments were repeated three times (n = 3). Source data are provided as a Source Data file.

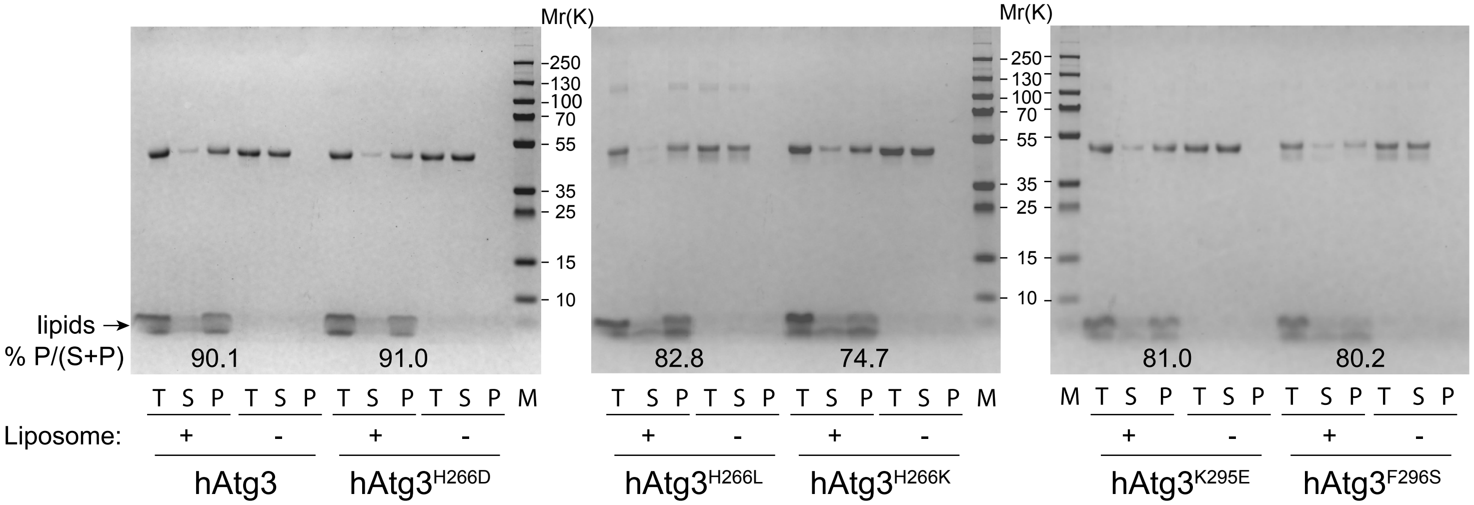

Supplementary Figure 9: Interaction of hAtg3 and its mutants with liposomes in co-sedimentation assay.

hAtg3 and its mutants (2 µM) were incubated with (+) and without (-) liposomes (800 µM) at 37 °C for 1 hr. Liposome-associated hAtg3 and its mutants were pelleted down by ultracentrifugation and analyzed by Sure-PAGE (10% Bis-Tris, Genscript). T: total; S: supernatant; P: pellet. The arrow indicates the lipid band. All experiments were repeated three times (n = 3). Source data are provided as a Source Data file.

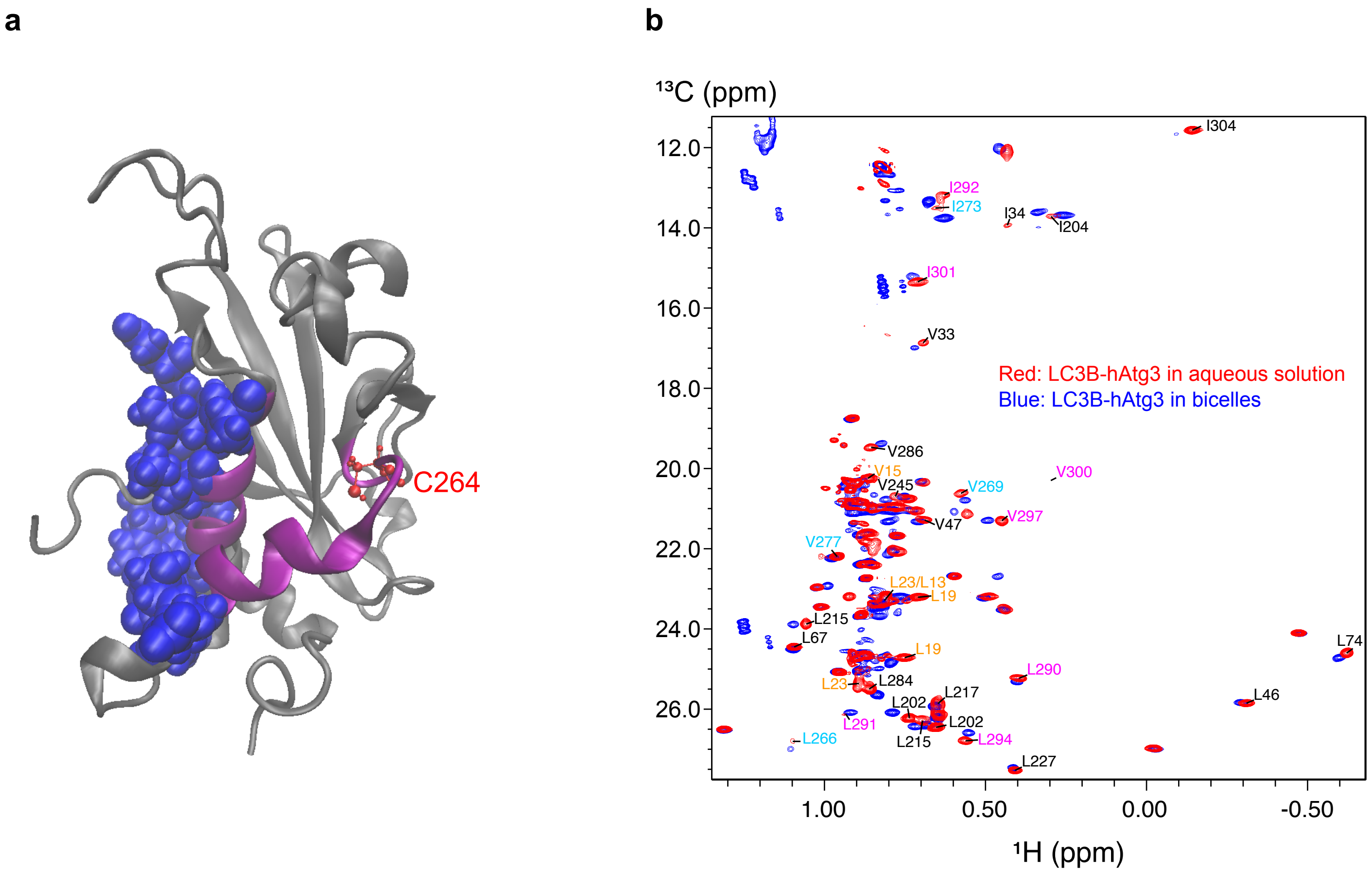

Supplementary Figure 10: The C-terminal regions of hAtg3 in the context of LC3B-hAtg3 interact with the membrane.

(a) The corresponding residues (blue) at the Atg8-yAtg3 interaction interface are mapped onto the hAtg3 structure. These residues include H45, L46, H48, H49, P51, E275, T276, V277, G285, H287, M288, K295, Q298, A299, E305, Y306, and D307. C-terminal regions that interact with the membrane are shown in purple. The active site Cys264 is shown in red.

(b) Overlay of ^{13}C-^{1}H HMQC spectra of perdeuterated, $^{13}CH_3$-ILV-labeled hAtg3$^{\Delta 90\text{-}190,\ H266L,\ C50A,\ C81A,\ C85A,\ C259A}$ linked to LC3B^{G120C} by a disulfide bond in aqueous solution (red) and in bicelles (blue, DMPC:DMPG:DHPC = 8:2:40, molar ratio). Perturbed residues are labeled with their assignments. Orange: residues from the N-terminal; cyan: C-terminal residues from 262 to 277; magenta: C-terminal residues from 290 to 301; black: residues from other regions. Spectra were acquired on a 600 MHz Bruker spectrometer at 25 °C, pH 7.5.

Supplementary Table 1 NMR and refinement statistics for hAtg3$^{\Delta 1\text{-}25,\ \Delta 90\text{-}190}$

	**Protein**
**NMR distance and dihedral constraints**	
Distance constraints	
Total NOE	2360
Intra-residue	633
Inter-residue	1727
Sequential ($\|i - j\| = 1$)	635
Medium-range ($\|i - j\| < 4$)	369
Long-range ($\|i - j\| > 5$)	723
Intermolecular	0
Hydrogen bonds	0
Total dihedral angle restraints	
ϕ	133
ψ	131
Total RDC restraints	
Phage	129
Negative gel	217
Neutral gel	92
Positive gel	114
**Structure statistics**	
Violations (mean and s.d.)	
Distance constraints (Å)	0.074 ± 0.003
Dihedral angle constraints (º)	0.838 ± 0.078
Max. dihedral angle violation (º)	7.1
Max. distance constraint violation (Å)	0.95
Deviations from idealized geometry	
Bond lengths (Å)	0.004 ± 0.000
Bond angles (º)	0.698 ± 0.022
Impropers (º)	0.605 ± 0.019
Q factor*	0.22 ± 0.01
Average pairwise r.m.s. deviation** (Å)	
Heavy	0.4
Backbone	0.9

*Definition of RDC Q factor is given in the literature (Cornilescu et al. J. Am. Chem. Soc., (1998), 120: 6836-6837.

**Pairwise r.m.s. deviation was calculated among 10 refined structures. Evaluated for secondary structure elements (excluding residues 268-278): 36-49, 54-56, 73-80, 199-207, 212-220, 229-232, 241-244, 245-248, 258-261, 289-297.

Supplementary Table 2 Primers (F, Forward; R, Reverse) used in this study.

Primers ID for in vitro experiments		Sequences from 5' to 3'
H266L	F	CCATGCAGGCTTGCTGAGGTG
	R	GTGAACTGAACACATGGGAG
H266K	F	CCCATGCAGGAAAGCTGAGGTGA
	R	TGAACTGAACACATGGGAGG
H240Y/V241A	F	CAGTCAGGATTATGCGAAGAAAACAG
	R	ATGTCTTCATACATGTGC
P263G/H266L	F	AGGCTTGCTGAGGTGATGAAGAAAATC
	R	GCATCCGTGAACTGAACACATGGG
K295E	F	TATTTTCTTGGAATTTGTACAAGC
K295E/L	R	AGAAGATACATATGAACTCC
K295L	F	TATTTTCTTGCTATTTGTACAAGCTGTC
F296L	F	TCTTGAAATTAGTACAAGCTGTC
	R	AAATAAGAAGATACATATGAACTC
F296K	F	TTTCTTGAAAAAAGTACAAGCTGTCATTC
F296E	F	TTTCTTGAAAGAAGTACAAGCTGTCATTC
F296A	F	TTTCTTGAAAGCTGTACAAGCTGTCATTC
F296K/E/A	R	ATAAGAAGATACATATGAACTCC
F296S	F	TTCTTGAAATCTGTACAAGCTG
	R	AATAAGAAGATACATATGAACTC
I274D	F	GAAGAAAATCGATGAGACTGTTGCAGAAGG
	R	ATCACCTCAGCATGCCTG
I273D	F	GATGAAGAAAGACATTGAGACTGTTGCAGAAGG
	R	ACCTCAGCATGCCTGCAT
K271D	F	TGAGGTGATGGACAAAATCATTGAGACTGTTGC
	R	GCATGCCTGCATGGGTGA
M270D	F	TGCTGAGGTGGATAAGAAAATCATTGAGACTGTTGCAGAAG
	R	TGCCTGCATGGGTGAACT
V269D	F	CATGCTGAGGACATGAAGAAAATCATTG
	R	CCTGCATGGGTGAACTGA
A267D	F	TGCCTGCATGGGTGAACT

	R	TGGGTGAACTGAACACATG
H266D	F	CCCATGCAGGGATGCTGAGGT
	R	TGAACTGAACACATGGGAGGTG
R265D	F	TGAACTGAACACATGGGAGGTG
	R	ACTGAACACATGGGAGGTG
C50A	*	ATCACCTAGTCCACCACGCTCCAACATGGCAATGGG
C81A_C85A	*	TGGTAACCAAAAATGTGCCGGCCTATAAGCGGGCCAAACAGATGGAATATTCAG
C259A	*	GCCACCACCTCCCATGGCTTCAGTTCACCCATGC

* These primers are designed for the construction of hAtg3$^{\Delta 90\text{-}190,\ H266L,\ C50A,\ C81A,\ C85A,\ C259A}$ with QuikChange Multi Site-Directed Mutagenesis Kit (Agilent).
